# OTUD7B suppresses Smac mimetic-induced lung cancer cell invasion and migration via deubiquitinating TRAF3

**DOI:** 10.1186/s13046-020-01751-3

**Published:** 2020-11-16

**Authors:** Boxiang Zhang, Chengcheng Yang, Rui Wang, Jie Wu, Yunfeng Zhang, Dapeng Liu, Xin Sun, Xiang Li, Hong Ren, Sida Qin

**Affiliations:** 1 Department of Thoracic Surgery, The First Affiliated Hospital of Xi’an Jiaotong University, 277 West Yanta Road, Xi’an, 710061 Shaanxi China; 2 Department of Oncology, The First Affiliated Hospital of Xi’an Jiaotong University, 277 West Yanta Road, Xi’an, 710061 Shaanxi China

**Keywords:** Smac mimetic, Llung cancer, OTUD7B, NF-κB pathway, LCL161

## Abstract

**Background:**

Smac mimetics are a type of drug that can induce apoptosis by antagonizing IAP family members in cancer treatment. However, a recent study showed that Smac mimetics can trigger cell invasion and migration in cancer cells by activating the NF-κB pathway.

**Methods:**

We assessed lung cancer cell elongation, invasion and migration under treatment with the Smac mimetic LCL161. Functional analyses (in vitro and in vivo) were performed to detect the contribution of NIK and OTUD7B to LCL161-induced cell invasion and migration. The role of OTUD7B in regulation of the TRAF3/NIK/NF-κB pathway under LCL161 treatment was analysed by immunoblotting, immunoprecipitation, luciferase and ubiquitin assays, shRNA silencing and plasmid overexpression. Expression levels of OTUD7B, NIK and TRAF3 in tissue samples from lung cancer patients were examined by immunohistochemistry.

**Results:**

We found that LCL161 stimulates lung cancer cell elongation, invasion and migration at non-toxic concentrations. Mechanistically, LCL161 results in NIK accumulation and activates the non-canonical rather than the canonical NF-κB pathway to enhance the transcription of target genes, such as IL-2 and MMP-9. Importantly, knockdown of NIK dramatically suppresses LCL161-induced cell invasion and migration by reducing the proteolytic processing of p100 to p52 and target gene transcription. Interestingly, we discovered that OTUD7B increases TRAF3 and decreases NIK to inhibit the non-canonical NF-κB pathway and that overexpression of OTUD7B suppresses LCL161-induced cell invasion and migration. Notably, OTUD7B directly binds to TRAF3 rather than to NIK and deubiquitinates TRAF3, thereby inhibiting TRAF3 proteolysis and preventing NIK accumulation and NF-κB pathway activation. Furthermore, the OTU domain of OTUD7B is required for the inhibition of LCL161-induced cell invasion and migration, as demonstrated by transfection of the C194S/H358R(CH) mutant OTUD7B. Finally, we investigated whether OTUD7B inhibits LCL161-induced lung cancer cell intrapulmonary metastasis in vivo, and our analysis of clinical samples was consistent with the above findings.

**Conclusions:**

Our study highlights the importance of OTUD7B in the suppression of LCL161-induced lung cancer cell invasion and migration, and the results are meaningful for selecting lung cancer patients suitable for LCL161 treatment.

**Supplementary Information:**

The online version contains supplementary material available at 10.1186/s13046-020-01751-3.

## Background

Lung cancer is one of the most aggressive malignancies and the leading cause of morbidity and mortality worldwide [1]. Non-small cell lung cancer (NSCLC), the most common type of lung cancer, accounts for 85–90% of all lung cancers [2]. Most lung cancer patients are diagnosed with locally advanced or metastatic disease. Despite recent improvements in chemotherapy, radiotherapy, targeted therapies and immunotherapy, the overall 5-year survival rate of NSCLC remains below 20% [3]. Tumour invasion, migration and apoptotic resistance are the predominant causes of recurrence and treatment failure in patients with NSCLC [4, 5].

Inhibitors of Apoptosis Proteins (IAPs) are essential regulators of apoptotic resistance and are frequently overexpressed in lung cancer [6]. Additionally, IAPs are related to poor prognosis in NSCLC and are suitable targets for cancer therapy [7]. Smac mimetics are a type of drug that can induce apoptosis by antagonizing IAP family members in cancer cells [8, 9]. Our previous study showed that the Smac mimetic LCL161 increases paclitaxel-induced apoptosis in NSCLC by degrading cIAP1 and cIAP2 [10]. In addition, many Smac mimetics have already been tested in early clinical trials [11, 12]. However, recent reports show that BV6, a Smac mimetic, can trigger cell elongation, invasion and migration in GBM cells at non-lethal concentrations [13, 14]. In addition, BV6 induces bone metastasis by altering the microenvironment [15]. This effect may be caused by activation of the non-canonical NF-κB pathway, which promotes the transcription of pro-invasive and migratory genes, such as matrix metalloproteinases (MMPs) and CXC chemokines [16, 17]. In contrast to the canonical NF-κB pathway, the non-canonical pathway selectively responds to a specific group of stimuli and is regulated by NF-κB inducing kinase (NIK) and ubiquitin-mediated proteasomal degradation of TRAF3 [18, 19]. Although LCL161 has been proven to be safe and well tolerated in phase 1 and phase 2 clinical trials, it remains unknown whether it triggers invasion and migration in lung cancer cells, and if so, how to prevent these events needs to be investigated.

OTU domain-containing 7B (OTUD7B) is a member of the OTU family that controls many important cell signalling pathways, such as those related to scar formation, inflammation and hypoxia regulation [20–22]. A previous study suggested that OTUD7B negatively regulates TRAF3 degradation by mediating its deubiquitination, thereby suppressing activation of the non-canonical NF-κB signalling pathway [23]. However, whether OTUD7B can inhibit LCL161-induced invasion and migration and be used to select a target population in LCL161 treatment for lung cancer is unclear.

In the present study, we demonstrate that LCL161 can induce invasion and migration in lung cancer cells at non-toxic concentrations. In addition, we found that LCL161-induced invasion and migration occurs through the non-canonical NF-κB pathway as well as activation of IL-2 and MMP-9. Moreover, OTUD7B inhibits LCL161-induced invasion and migration by binding to and deubiquitinating TRAF3, thereby inhibiting NIK and preventing non-canonical NF-κB activation. DUB activity of OTUD7B is necessary for inhibition of non-canonical NF-κB activation and LCL161-induced invasion and migration. Finally, the conclusions above were verified in animal experiments, and the results of clinical sample analysis were consistent with the above. Our study provides theoretical and experimental evidence for the selection of appropriate patients and more effective individualized targeted therapy.

## Methods

### Cell culture and drug treatment

Human lung cancer cell lines A549, H460, H1299, H157, and H1944 were obtained from American Type Culture Collection (ATCC, VA, USA). A549, H460, H157, and H1944 cells were cultured in DMEM and H1299 cells in RPMI-1640 medium (Life Technologies, Inc., Eggenstein, Germany) at 37 °C in 5% CO_2_. All media were supplemented with 1% penicillin/streptomycin (Invitrogen) and 10% foetal calf serum (Invitrogen). For drug treatment, cells were seeded in 6- or 96-well plates and treated with various concentrations of LCL161.

### Plasmids, transfection and infection

HA-tagged OTUD7B, OTUD7B C194S/H358R, Flag-tagged TRAF3 and ubiquitin vectors were a kind gift from Prof. Shaocong Sun. Myc-NIK and Flag NIK were constructed by cloning the corresponding cDNAs into the pcDNA3-Myc or pcDNA3-Flag vector using BamHI and EcoRI sites. Lentiviral shRNA vectors for depleting human NIK were purchased from Thermo Fisher Scientific (Dreieich, Germany). The PCLXSN-HA-OTUD7B or control plasmid was transfected into 293 T cells with CaCl_2_, VSVG and Ampho. Lentiviral particles were prepared by transfecting 293 T cells with pGIPZ vectors encoding shNIK or control shRNA along with packaging plasmids. (The sequences of shNIK are shown in Table S1). Two days after transfection, lentiviral supernatants were harvested and used to infect certain cells. After incubation for 24 h at 37 °C, the medium containing lentiviral particles was removed, and fresh medium was added. The infected cells were selected by flow cytometry or antibiotic and used for further experiments.

### Determination of cell elongation

Cells were seeded in 12-well plates before stimulation. After stimulation with LCL161 for 24 h, live cells imaging was aquired by using microscope (Leica Microsystems, Wetzlar, Germany). Three pictures of each condition were selected. Cell length and width of 10 cells in pictures were measured using ImageJ (Bethesda, MD, USA). Cell elongation index was calculated by dividing the length by the width.

### Immunoprecipitation and immunoblotting

Immunoprecipitation assays and western blotting were performed as described previously [24] using the following antibodies: anti-OTUD7B, anti-cIAP-1, anti-cIAP-2, XIAP, anti-p100/p52, anti-cleaved caspase-3, anti-RelB, anti-p-IκB-α, anti-IκB-α, anti-NIK, anti-TRAF3, anti-HA, anti-Flag. β-Actin was used as a loading control. All antibodies used in the study are shown in Table S2. Cytosolic and nuclear fractionations were performed according to manufacturer’s protocol (Active Motif, #40010).

### Quantitative real-time RT-PCR

Total RNA was extracted and reverse transcribed using a PrimeScript RT reagent Kit (Takara, Beijing, China, Cat# RR047A). Quantitative real-time PCR was performed with FastStart Universal SYBR Green Master Mix (Roche, Cat# 04194194001) according to the manufacturer’s instructions. Relative quantification of each target gene was normalized by using an endogenous control (β-Actin). The primer sequences for all genes are shown in Table S1.

### Determination of cell viability

Cell viability was assessed using the 3-(4,5-dimethylthiazol-2-yl)-2,5-diphenyltetrazolium bromide (MTT) assay based on the manufacturer’s instructions (Roche Diagnostics, Mannheim, Germany).

### Scratch wound-healing assay

Cells were seeded in 6-well plates (Corning, NY, USA) and allowed to grow to full confluence. The cells were scratched with a 100-μl pipette tip, and migration distances were measured at 0, 24 h, and 48 h after scratching. Five fields were randomly selected for each experiment.

### Transwell assay

A total of 2 × 10^4^ cells were seeded onto 8-μm chambers (Corning, Wiesbaden, Germany), and the indicated concentration of LCL161 were added to both the lower and upper chambers for 24 h for stimulation. Cells on the upper chambers were scraped off using a cotton swab, and the cells in the lower chamber were fixed in 4% paraformaldehyde and stained with 0.4% crystal violet. The cells were quantified by inverted microscopy.

### Luciferase assay

Cells were seeded in 12-well plates and transfected with the 3 kB-firefly luciferase vector and Renilla luciferase vector. After transfection, the cells were treated with the indicated concentration of LCL161 or DMSO. After 24 h of stimulation, the cells were lysed, and a microplate reader was used for measurements. Luciferase values were normalized to Renilla luciferase values.

### CHX chase assay

For the cycloheximide (CHX) chase assay, CHX (Sigma-Aldrich, St Louis, USA) at a concentration of 50 mg/ml was added to the medium; the cells were harvested at the indicated times (0, 1, 2, 4, 6 h) and prepared for Western blotting assays with anti-HA, anti-Flag, and anti-actin antibodies. MG132 (Enzo Life Sciences, Lorrach, Germany) was used as a positive control.

### Ubiquitination assay

For the ubiquitination assay, Flag-TRAF3, HA-OTUD7B, Myc-NIK and Ub were transfected into H1299 cells, and cell lysates were immunoprecipitated with an anti-Flag antibody. The level of Flag-TRAF3 ubiquitination was detected by western blotting.

### In vivo experiment

Four-week-old BALB/c nude mice were purchased from the Laboratory Animal Center of Xi’an Jiaotong University. All mice were bred under specific-pathogen-free conditions. All animal experiments were approved by the Ethics Committee of the First Affiliated Hospital of Xi’an Jiaotong University.

To investigate whether OTUD7B can inhibit LCL161 induced-invasion and metastasis in vivo, four groups of six mice each were administered 5 × 106 cells by intravenous tail vein injections. The control and LCL161 groups were injected via the caudal vein with empty vector stably transfected H1299 cells. The OTUD7B(WT) and OTUD7B(CH) groups were injected with OTUD7B(WT)-overexpressing H1299 cells and OTUD7B(CH)-overexpressing H1299 cells, respectively. We treated the groups except the control group with 2 mg/kg LCL161 through intraperitoneal injection every 2 days for 3 weeks. After 4 weeks, the mice were sacrificed, and their body weights were recorded every 2 days. Lung tissues were excised and embedded in paraffin for further study.

To further verify the mechanism of OTUD7B inhibiting LCL161-induced invasion and metastasis in vivo, a total of 5 × 10^6^ H1299 cells with stably transfected EV, OTUD7B(WT) and OTUD7B(CH) were subcutaneously injected into the right flank of nude mice. When the average tumor volume reached 100 mm^3^, we treated the mice with 2 mg/kg LCL161 through intraperitoneal injection every 2 days for 3 weeks except for the control group. After the mice were sacrificed, all tumors were embedded in paraffin for hematoxylin and eosin staining and immunohistochemistry.

### Patient characteristics

All specimens were obtained from 146 NSCLC patients who were diagnosed and underwent curative resection between March 2006 and April 2013 at the First Affiliated Hospital of Xi’an Jiaotong University. The TNM stage was reconfirmed with original information according to the eighth edition of the TNM classification system of the International Association for the Study of Lung Cancer in 2016. Overall survival was defined as the time from resection to death or the last follow-up. The last follow-up date was June 2017. The median follow-up period was 41 months (4 to 120 months). The main cause of death was NSCLC recurrence. The characteristics of NSCLC patients are shown in Table S3. The study was approved by the Ethics Committee of the First Affiliated Hospital of Xi’an Jiaotong University, based on the patients’ written informed consent for the usage of the biologic material.

### IHC staining

IHC staining was performed as described previously [10]. Briefly, tissue slides were pre-treated and incubated with monoclonal antibodies against OTUD7B (1:400 dilution, Proteintech), TRAF3 (1:1000 dilution, Cell Signalling) and NIK (1:400 dilution, Cell Signalling) monoclonal antibodies overnight at 4 °C. After washing with PBS, the slides were incubated with the indicated secondary antibody and stained with haematoxylin.

Both the extent and intensity of staining were taken into consideration. The extent of staining was scored from 0 to 100% (1 indicated 1–25% stained, 2 indicated 26–50% stained, 3 indicated 51–75% stained, 4 indicated 76–100% stained), and the staining intensity was scored from 0 to 2 (0 indicated no staining, 1 indicated weak to moderate staining, 2 indicated strong staining). The IHC score was the sum of the staining extent score and intensity score. The IHC score was used to define the level of expression as follows: high expression (+): score ≥ 3; low expression (−): score ≤ 2.

### Statistical methods

Statistical analysis was performed using two-sided standard Student’s t-tests or the χ2 test, and differences with probability values of *P* < 0.05 were regarded as statistically significant. Spearman correlation analysis was used to analyse the correlation of OTUD7B, TRAF3 and NIK status. The analysis was conducted using GraphPad Prism v.6.0c (GraphPad Software) and the R package (× 64, 3.6.1).

## Results

### LCL161 stimulates lung cancer cell elongation, invasion and migration at non-toxic concentrations

**Fig. 1:**
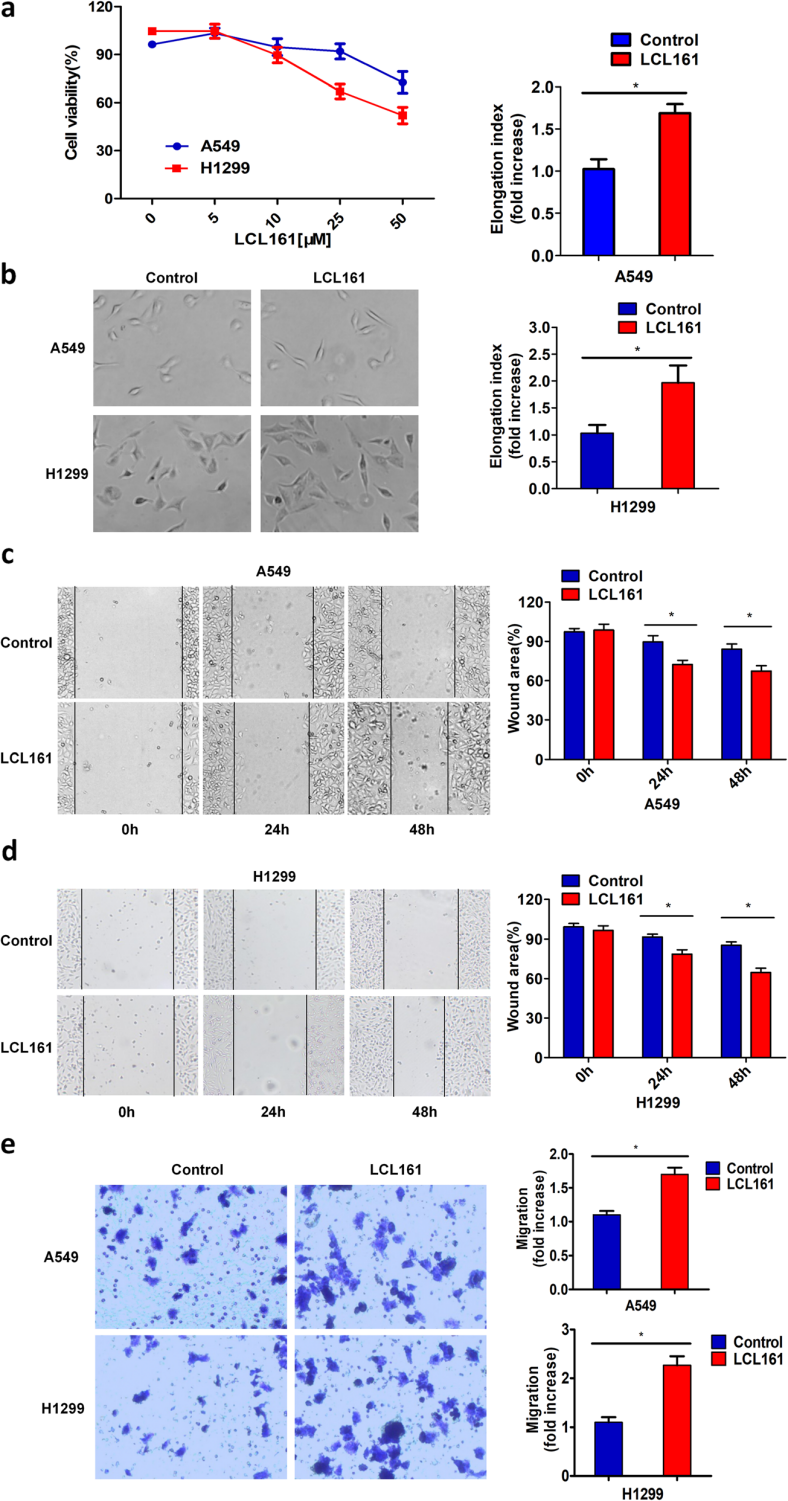
LCL161 triggers lung cancer cell elongation, invasion and migration at a nontoxic concentration. **a** A549 and H1299 cells were treated with the indicated concentration of LCL161 or DMSO for 24 h. Cell viability was detected by the MTT assay. **b** H1299 / A549 cells were treated with 10 μM / 25 μM LCL161 or DMSO for 24 h. Cell morphology was analysed by phase-contrast microscopy. The cell elongation index (length/width) was determined by measuring cell length and width. Magnification, × 100. *P < 0.05. **c**, **d** A549 and H1299 lung cancer cells were plated and allowed to grow to confluence. The medium was removed, and wounds in the confluent monolayers were produced with a 100-μl pipette tip. After treatment with the indicated concentrations of LCL161 or DMSO for 24 h or 48 h, the wound-healing process was monitored by an inverted light microscope. **e** A549 and H1299 lung cancer cells were treated with the indicated concentrations of LCL161 or DMSO for 48 h. The migrated cells in a collagen-coated transwell migration chamber were fixed and stained with crystal violet, and optical density was measured. Magnification, × 100. Representative results are shown. Plots are the mean ± SEM of data from three independent experiments. *P < 0.05

To examine whether the Smac mimetic LCL161 at a non-toxic concentration can induce invasion and migration in lung cancer cells, we initially needed to define the non-lethal concentrations to avoid the effect of apoptosis on subsequent assays. LCL161 had no apparent effect on the expression of XIAP and activated caspase-3, apoptosis rate or viability in A549 cells at concentrations up to 25 μM or in H1299 cells up to 10 μM. However, under these conditions, LCL161 could still trigger the degradation of cIAP1 and cIAP2 (Fig. 1a, S1a-c). Thus, we chose 25 μM LCL161 for A549 cells and 10 μM for H1299 cells as a non-toxic concentration for ensuing experiments.

To explore the effect of LCL161 on cell elongation, invasion and migration, we used phase-contrast microscopy to monitor the morphology of H1299 / A549 cells. Interestingly, we found that after treatment with 10 μM / 25 μM LCL161, the cells became tapered and had longer cytoplasmic extensions than control cells. Therefore, LCL161 stimulates lung cancer cell elongation at a non-toxic concentration (Fig. 1b). In wound-healing assays with the indicated concentrations of LCL161 or DMSO, we found that LCL161 significantly enhanced the invasion of A549 and H1299 cells at 24 h and 48 h (Fig. 1c, d). In addition, transwell migration assays revealed that treatment with LCL161 at 48 h increased the migration of A549 and H1299 cells (Fig. 1e).

#### LCL161 activates the non-canonical NF-κB pathway to enhance target gene transcription

**Fig. 2:**
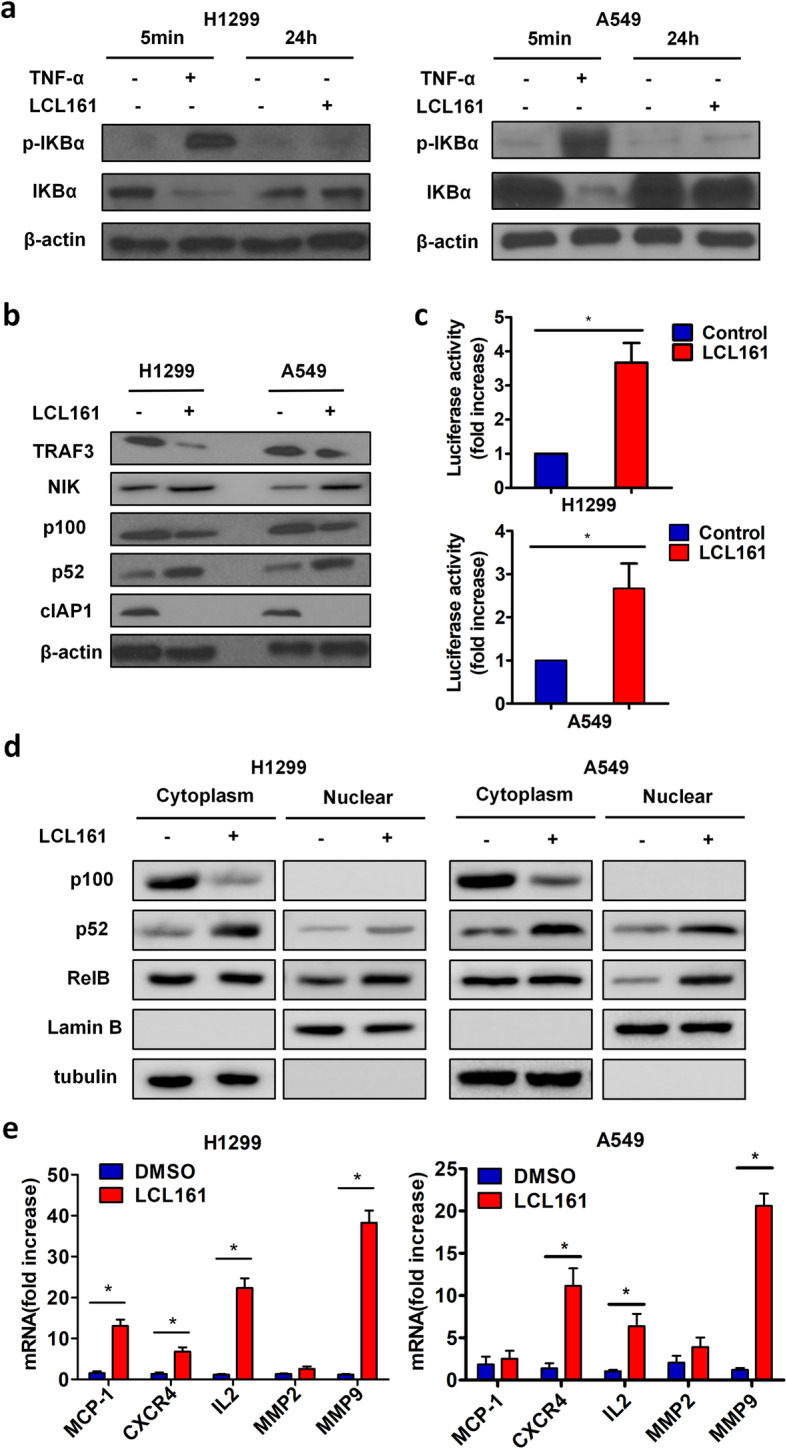
LCL161 activates the non-canonical NF-κB pathway to enhance target gene transcription. **a** H1299 and A549 cells were treated with 10 μM/25 μM LCL161 or DMSO for 24 h. Cells were stimulated with 10 ng/ml TNFα for 5 min as a positive control. Phosphorylation of IκBα and IκBα was analysed by Western blotting. **b** After cells were treated with LCL161 or DMSO for 24 h, the expression levels of TRAF3, NIK, p100, p52, and cIAP1 were analysed by Western blotting. β-Actin served as the loading control. **c** NF-kB transcriptional activity was assessed by a luciferase assay, and the fold increase in luciferase activity is shown. **d** The expression of p100, p52 and RelB in both cytoplasm and nuclear were detected by Western blotting. Lamin B and tubulin served as the loading control. **e** The mRNA levels of some NF-κB target genes were examined by quantitative RT-PCR. Representative results are shown. Plots are the mean ± SEM of data from three independent experiments. *P < 0.05

To identify the molecular mechanisms by which LCL161 induces cell elongation, invasion and migration, we examined the effect of LCL161 on both the canonical and non-canonical NF-κB pathways. After H1299 and A549 cells were treated with 10 μM and 25 μM LCL161, respectively, for 24 h, we detected IkBα by Western blotting and found that it was not phosphorylated. As a positive control, we used 10 ng/ml TNF-α to stimulate cells and observed IkBα phosphorylation (Fig. 2a). Thus, the canonical NF-κB pathway was inactivate at the indicated concentrations of LCL161. However, for the non-canonical NF-κB pathway, LCL161 significantly decreased the expression of cIAP1, leading to the accumulation of NIK and proteolytic processing of p100 to p52. Importantly, LCL161 also decreased TRAF3 expression (Fig. 2b). To better explore the molecular mechanism in which the LCL161 increases NF-κB transcriptional activity, we performed nuclear translocation assay for non-canonical NF-κB pathway analysis. After treatment with LCL161 for 24 h, the expression of p100, p52 and RelB in both cytoplasm and nuclear were detected by Western blotting. We found that LCL161 induced processing of p100 to p52 in cytoplasm and boosted nuclear translocation of p52 in H1299 and A549 cells (Fig. 2c). To explore whether LCL161 impact NF-κB transcription activity, a luciferase assay was applied by using luciferase reporter plasmid containing NK-κB binding sequence. And we observed LCL161 significantly increases NF-κB transcription activity (Fig. 2d). In addition, LCL161 increased the mRNA level of NF-κB target genes that control cell invasion and migration, including monocyte chemoattractant protein 1 (MCP-1), CXC chemokine receptor type 4 (CXCR4), interleukin-2 (IL-2) and matrix metallopeptidase 9 (MMP9) (Fig. 2e). This set of experiments demonstrates that LCL161 causes only non-canonical NF-kB activation and enhances NF-kB target gene transcription in lung cancer cells.

#### NIK is required for LCL161-induced NF-κB pathway activation and cell elongation, invasion and migration

**Fig. 3:**
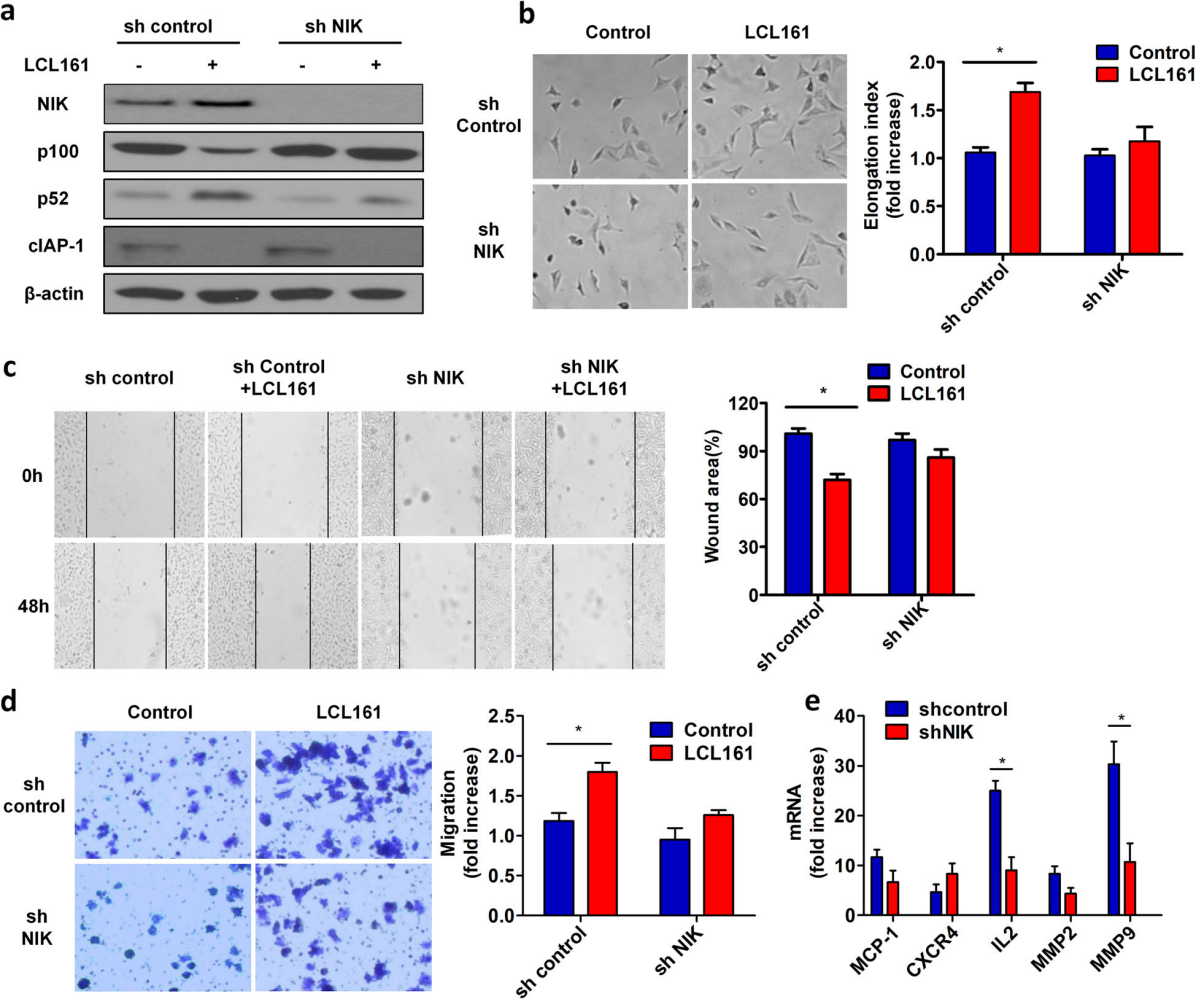
NIK is required for LCL161-induced NF-κB pathway activation and cell elongation, invasion and migration. H1299 cells were transfected with shRNA against NIK or the control vector for 48 h and then treated with 10 μM LCL161 or DMSO for 24 h. **a** Expression of NIK, p100, p52, and cIAP-1 was analysed by Western blotting. β-Actin was used as a loading control. **b** Cell morphology was analysed by phase-contrast microscopy. Magnification, × 100. Cell elongation was quantified by measuring cell length and width and by calculating the cell elongation index (length/width), and the fold increase in the elongation index is shown. **c** The wound-healing process was monitored by an inverted light microscope. **d** Cells were plated in a collagen-coated transwell migration chamber. After treatment, the migrated cells were fixed and stained with crystal violet, and optical density was measured. **e** The mRNA levels of NF-κB target genes were evaluated by quantitative RT-PCR. Representative results are shown, and plots represent the mean ± SEM of data from three independent experiments. *P < 0.05

NIK is the key component in the non-canonical NF-kB pathway. To further explore the role of this pathway in LCL161-induced cell elongation invasion and migration, we generated NIK-knockdown H1299 cells by transfection with an shRNA plasmid targeting NIK. LCL161-induced NIK accumulation was reduced by NIK silencing, and less p100 was processed to p52 than in the shRNA control group (Fig. 3a). In addition, knockdown of NIK dramatically reduced LCL161-induced cell elongation, invasion and migration compared to that in the control group (Fig. 3b-d). LCL161-mediated upregulation of IL-2 and MMP9 mRNA expression was significantly reduced in NIK-knockdown cells (Fig. 3e). These experiments show that NIK is required for LCL161-induced cell elongation, invasion and migration, as well as for activation of the non-canonical NF-kB pathway.

#### OTUD7B inhibits NIK to suppress LCL161-induced NF-κB pathway activation and cell elongation, invasion and migration

**Fig. 4:**
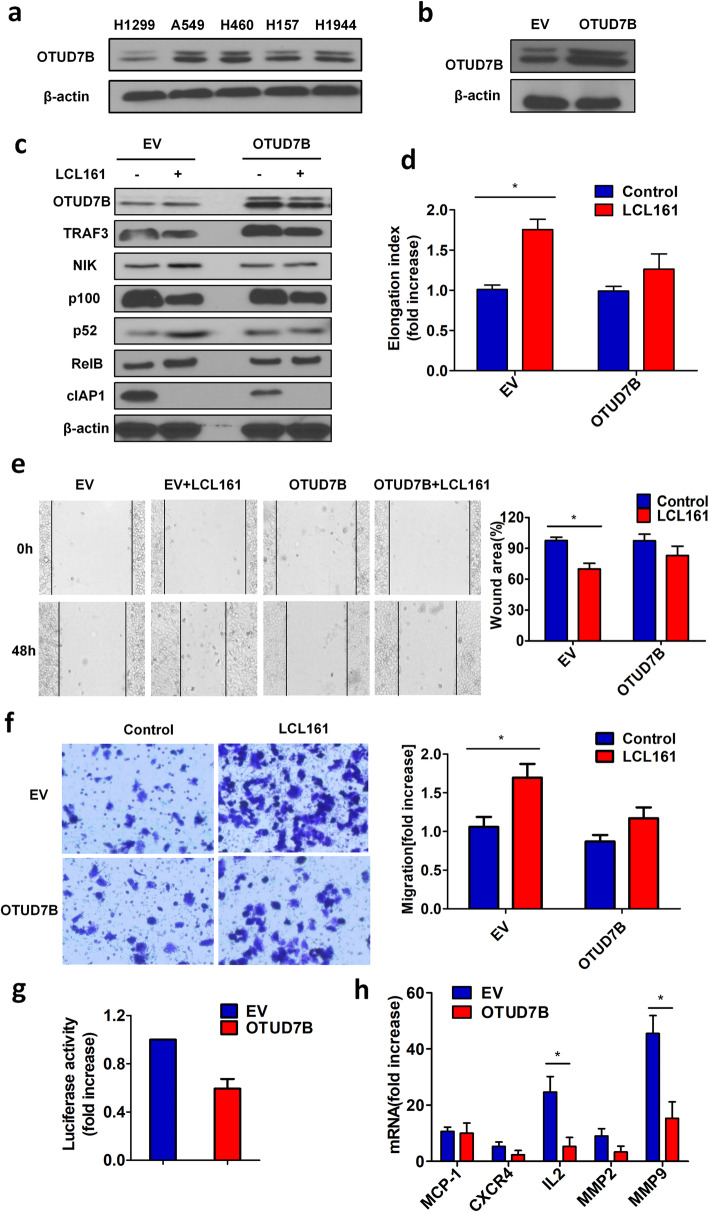
OTUD7B inhibits NIK to suppress LCL161-induced NF-κB pathway activation and cell elongation, invasion and migration. **a** Expression of OTUD7B was determined in several lung cancer cell lines, including H1299, A549, H460, H157, and H1944 cells. **b** H1299 cells were stably transfected with pcDNA-HA-OTUD7B or empty vector and confirmed by Western blotting. **c**-**h** OTUD7B-overexpressing H1299 cells and control cells were treated with 10 μM LCL161 or DMSO for 24 h. **c** Expression of TRAF3, NIK, p100, p52, and cIAP1 was measured by Western blotting. β-Actin was used as a loading control. **d** Cell elongation was quantified by calculating the cell elongation index (length/width); the fold increase in the elongation index is shown. **e** The wound-healing process was monitored by an inverted light microscope. **f** Cells were plated in a collagen-coated transwell migration chamber. After treatment, the migrated cells were fixed and stained with crystal violet, and optical density was measured. **g** NF-kB transcriptional activity was analysed by a luciferase assay, and the fold increase in luciferase activity is shown. **h** mRNA levels of NF-κB target genes were examined by quantitative RT-PCR. Representative results are shown. Plots are the mean ± SEM of data from three independent experiments. *P < 0.05

To investigate how OTUD7B inhibits LCL161-induced cell elongation, invasion and migration, we established H1299 cells that overexpressed OTUD7B. First, we assessed the protein expression of OTUD7B protein in several lung cancer cells and found that it was lowest in H1299 cells (Fig. 4a). OTUD7B was stably transfected into H1299 cells, as revealed by Western blotting (Fig. 4b). OTUD7B increased expression of TRAF3, leading to a decrease in NIK and processing of p100 to p52 compared to those in the empty vector group (Fig. 4c). In addition, OTUD7B overexpression prevented LCL161-mediated cell elongation, invasion and migration after treatment with LCL161 (Fig. 4d-f). OTUD7B overexpression also inhibited LCL161-mediated upregulation of NF-κB transcriptional activity (Fig. 4g). Importantly, LCL161-mediated MMP9 and IL-2 were profoundly suppressed in OTUD7B-overexpressing cells (Fig. 4h). These experiments indicate that overexpressed OTUD7B inhibits LCL161-induced cell elongation, invasion, migration and induction of NF-κB target genes.

#### OTUD7B deubiquitinates TRAF3 to inhibit NIK

**Fig. 5:**
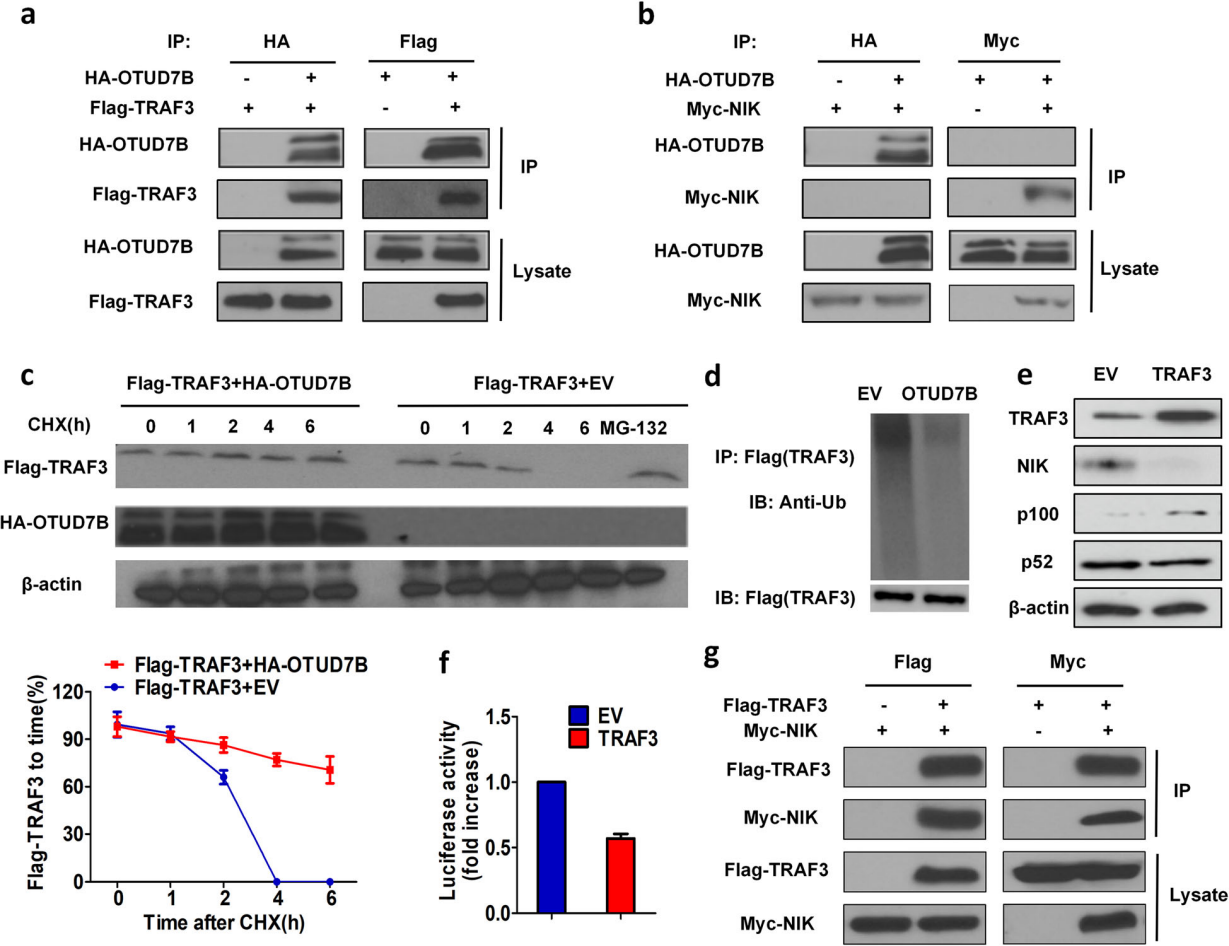
OTUD7B deubiquitinates TRAF3 to inhibit NIK. **a** Flag-TRAF3 and HA-OTUD7B plasmids were cotransfected into H1299 cells. TRAF3 or OTUD7B was immunoprecipitated with an anti-Flag or anti-HA antibody. **b** Myc-NIK and HA-OTUD7B plasmids were cotransfected into H1299 cells. NIK or OTUD7B was immunoprecipitated with an anti-Myc or anti-HA antibody. **c** H1299 cells were transfected with Flag-TRAF3 and HA-OTUD7B or the empty vector. The half-life of TRAF3 was assessed following treatment with cycloheximide (CHX) for 0–6 h. MG132 was applied to inhibit TRAF3 degradation as a positive control. Cell lysates were analysed by using anti-Flag and anti-HA antibodies. **d** H1299 cells were transfected with Flag-TRAF3, Ub and HA-OTUD7B or the empty vector. The ubiquitin assay was performed by Western blotting using an anti-ubiquitin antibody. **e** H1299 cells were transfected with Flag-TRAF3 or the empty vector for 48 h and then treated with 10 μM LCL161 for 24 h. Expression of TRAF3, NIK, p100 and p52 was examined by Western blotting. β-Actin expression served as a loading control. **f** NF-kB transcriptional activity was analysed by a luciferase assay, and the fold increase in luciferase activity is shown. **g** Flag-TRAF3 and Myc-NIK plasmids were cotransfected into H1299 cells. TRAF3 or NIK was immunoprecipitated with an anti-Flag or anti-Myc antibody. Representative results are shown. Plots are the mean ± SEM of data from three independent experiments.*P < 0.05

To examine the potential mechanism by which OTUD7B inhibits the LCL161-induced NF-κB pathway, we transfected Flag-TRAF3 and HA-OTUD7B, Myc-NIK and HA-OTUD7B into H1299 cells for 48 h. Reciprocal co-immunoprecipitation was performed using anti-Flag anti-Myc and anti-HA antibodies to confirm whether OTUD7B directly binds to NIK or TRAF3. OTUD7B did not bind to NIK directly, but it did bind to TRAF3 (Fig. 5a, b). Moreover, a CHX chase assay was performed by treatment with a protein synthesis inhibitor, cycloheximide (CHX), for 0–6 h to confirm OTUD7B-mediated prevention of TRAF3 degradation in H1299 cells. At 4 h, the half-life of TRAF3 was significantly decreased in control cells compared with co-transfected cells (Fig. 5c). A ubiquitin assay was performed using an anti-ubiquitin antibody in H1299 cells transfected with Flag-TRAF3 and HA-OTUD7B or the empty vector, and OTUD7B overexpression drastically decreased TRAF3 ubiquitin levels (Fig. 5d). We also transfected Flag-TRAF3 and Myc-NIK into H1299 cells and found that after reciprocal co-immunoprecipitation, TRAF3 directly bound to NIK and inhibited the expression of NIK and NF-κB transcriptional activity (Fig. 5e-g).

#### DUB activity of OTUD7B is required for inhibition of LCL161-induced cell elongation, invasion and migration

**Fig. 6:**
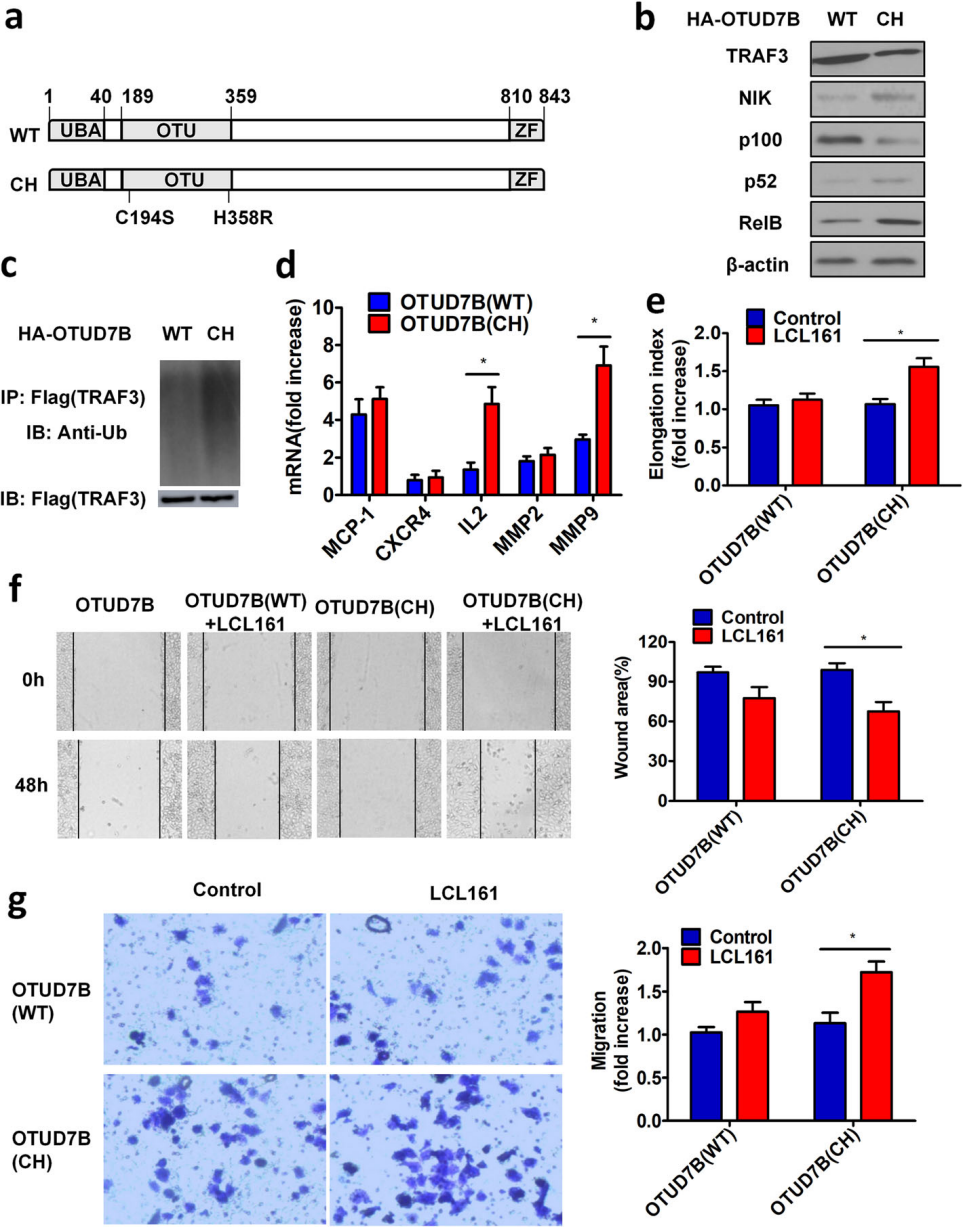
DUB activity of OTUD7B is required for inhibition of LCL161-induced cell elongation, invasion and migration. **a** A C194S/H358R(CH) mutant in the OTU domain was produced using the HA-OTUD7B plasmid. **b** H1299 cells were transfected with pcDNA-HA-OTUD7B(WT) or pcDNA-HA-OTUD7B(CH). After treatment with 10 μM LCL161 for 24 h, the cells were analysed for expression of TRAF3, NIK, p100, p52, and RelB by Western blotting. β-Actin expression served as a loading control. **c** H1299 cells were transfected with Flag-TRAF3 and HA-OTUD7B (WT) or Flag-OTUD7B (CH). After treatment with the indicated concentrations of LCL161, a ubiquitin assay in H1299 cells was performed by Western blotting using an anti-ubiquitin antibody. **d**-**g** H1299 cells were transfected with Flag-TRAF3 and HA-OTUD7B or Flag- OTUD7B(CH) and treated with the indicated concentrations of LCL161 for 48 h. **d** mRNA levels of NF-κB target genes were examined by quantitative RT-PCR. **e** Cell elongation was quantified by calculating the cell elongation index (length/width); the fold increase in the elongation index is shown. **f** The wound-healing process was monitored by an inverted light microscope. **g** Migrated cells were placed in a collagen-coated transwell migration chamber, fixed and stained with crystal violet, and optical density was measured. Representative results are shown. Plots are the mean ± SEM of data from three independent experiments. *P < 0.05

To determine which region of OTUD7B plays a key role in the inhibition of LCL161-induced cell elongation, invasion and migration, we used the OTU domain mutant C194S/H358R(CH) in the HA-OTUD7B plasmid (Fig. 6a). We transfected HA-OTUD7B(WT) and HA-OTUD7B(CH) into H1299 cells and found that OTUD7B(CH) decreased the expression of TRAF3 and led to an increase in NIK and processing of p100 to p52 compared to that in the OTUD7B(WT) cells (Fig. 6b). Additionally, the OTUD7B(CH) mutant drastically increased TRAF3 ubiquitin levels (Fig. 6c). Furthermore, after treatment with the indicated concentrations of LCL161, the mRNA expression of IL-2 and MMP-9 were increased in OTUD7B(CH) mutant H1299 cells (Fig. 6d). Wound-healing and transwell migration assays showed restoration of LCL161-induced cell elongation, invasion and migration in OTUD7B(CH) mutant H1299 cells compared to OTUD7B(WT) H1299 cells (Fig. 6e-g).

### OTUD7B inhibits LCL161-induced lung cancer cell intrapulmonary metastasis in vivo

**Fig. 7:**
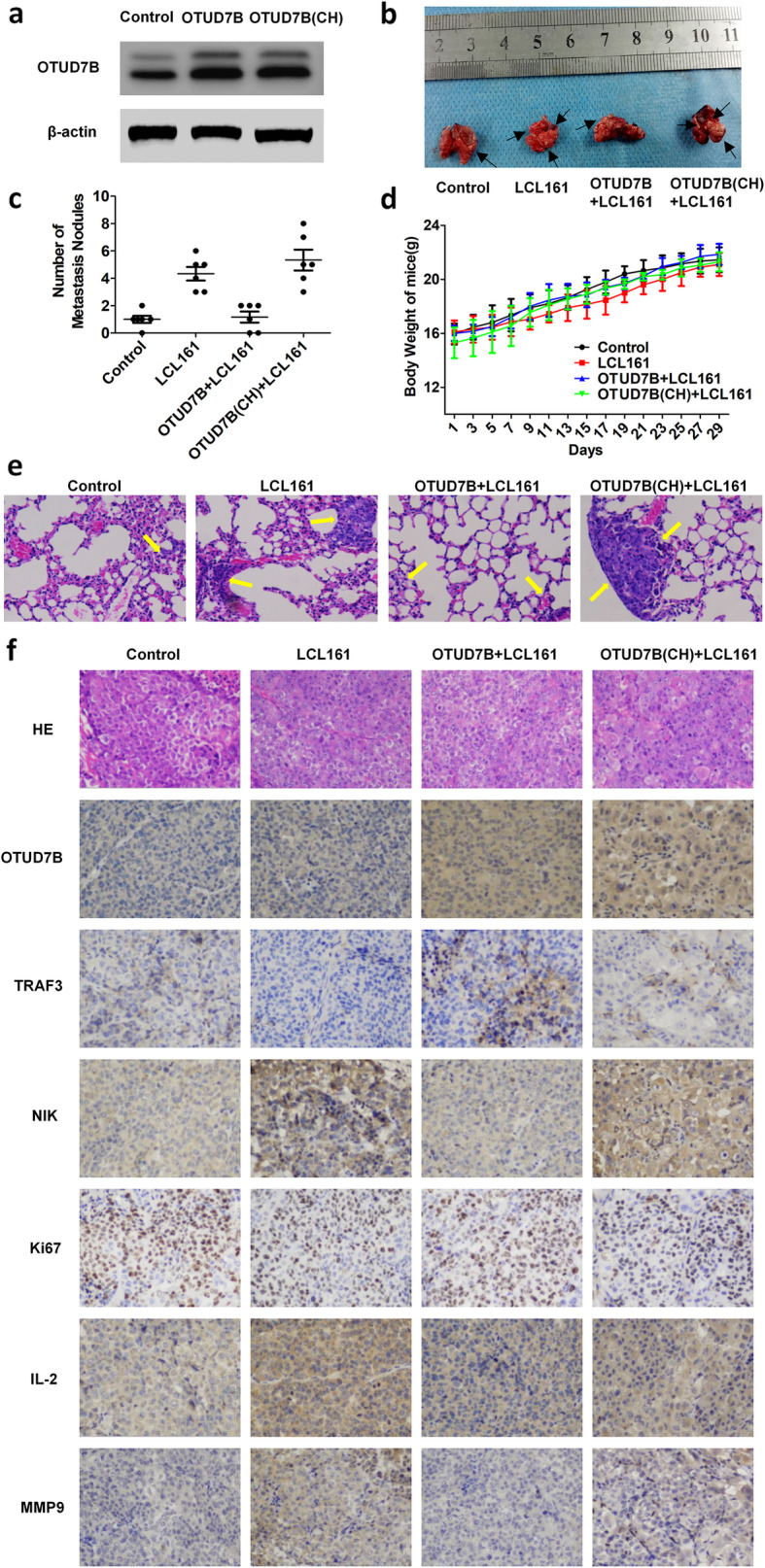
OTUD7B inhibits LCL161-induced lung cancer cell intrapulmonary metastasis in vivo. **a** Detection of OTUD7B expression in the stable cell lines by Western blotting. β-Actin expression served as a loading control. **b** Intrapulmonary metastasis was revealed by experiments in nude mice. Representative lungs of mice in different groups are shown. **c** HE staining was performed to detect the numbers of lung metastatic nodules. **d** The total number of intrapulmonary metastatic nodules in different groups is shown. *p < 0.05. **e** The body weights of the mice in the groups are provided. *P < 0.05

To confirm that OTUD7B inhibits LCL161-induced lung cancer cell metastasis in vivo, we stably transfected empty vector, HA-OTUD7B(WT) and HA-OTUD7B(CH) plasmids into H1299 cells (Fig. 7a) and implanted the cells into nude mice via caudal vein injection. We treated the mice with 2 mg/kg LCL161 through intraperitoneal injection every 2 days for 3 weeks except for the control group. After 4 weeks of growth, the mice were sacrificed, and the tumour nodules in lungs were examined by HE staining (Fig. 7b, c). As expected, there were more tumour nodules in the LCL161 group than in the control group but fewer tumour nodules in the OTUD7B + LCL161 group than in the LCL161 group. However, the tumour nodule numbers in the OTUD7B(CH) + LCL161 group were much greater than those in the OTUD7B + LCL161 group (Fig. 7d, e).

To further prove the mechanisms of OTUD7B inhibiting LCL161-induced invasion and metastasis in vivo, we performed IHC assay in the tumor nodules by using a subcutaneous tumor mode. We performed H&E staining to verify tumor tissues and immunohistochemistry to detect the expression of OTUD7B, TRAF3, NIK, Ki67, MMP9 and IL-2 in the xenografted tissues. As expected, LCL161 up-regulated the expression of NIK, MMP9 and IL-2 and down-regulated TRAF3 expression. However, OTUD7B rather than OTUD7B(CH) overexpression inhibited the up-regulation or down-regulation of these protein expression caused by LCL161 (Fig. 7f). Taken together, these data shows that LCL161 can induce lung cancer cell intrapulmonary metastasis in vivo and that metastasis is inhibited by OTUD7B but not by mutant OTUD7B, which lacks DUB activity. And the mechanisms of OTUD7B inhibiting LCL161-induced invasion and metastasis was also proved in vivo.

### Analysis of OTUD7B, TRAF3 and NIK in tissue samples from NSCLC patients and in clinical databases

**Fig. 8:**
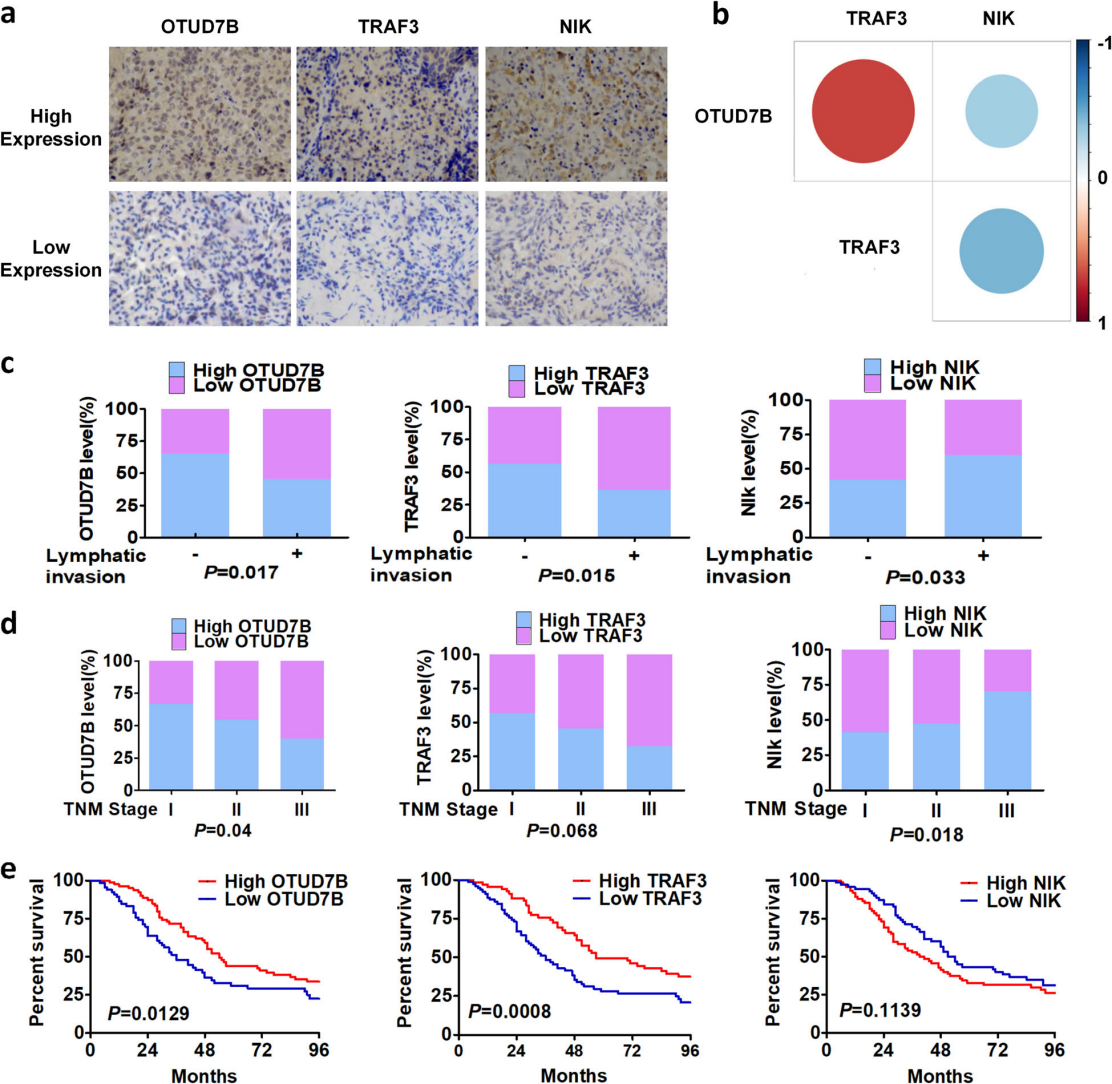
Analysis of OTUD7B, TRAF3 and NIK in tissue samples from NSCLC patients. **a** Representative images of immunohistochemistry (IHC) for OTUD7B, TRAF3 and NIK in cancer tissues from 146 clinical NSCLC patients (× 200). **b** Correlation analysis among OTUD7B, TRAF3 and NIK expression in human NSCLC specimens (*n* = 146). **c**, **d** The percentage of patients with high or low OTUD7B, TRAF3 and NIK expression in different groups divided by lymphatic invasion and TNM stage. **e** Kaplan-Meier survival curve of OTUD7B, TRAF3 and NIK proteins in NSCLC patients. Marks on the graph lines represent censored samples. The *P*-value refers to two-sided log-rank tests

To verify the clinical application value of our results, we analysed the expression of OTUD7B, TRAF3 and NIK in 146 lung cancer tissues and their correlation with prognosis. Representative images of immunohistochemistry (IHC) are shown in Fig. 8a. Correlation analysis among OTUD7B, TRAF3 and NIK expression in human NSCLC was performed, and Fig. 8b was produced using the R package. We found that high expression of OTUD7B was more frequently observed in early-stage lung cancer patients without lymph node metastasis. Indeed, low expression of TRAF3 in patients was related to lymph node metastasis. In contrast, high expression of NIK was more frequently observed in late-stage patients with lymph node metastasis (Fig. 8c, d). Furthermore, we discovered that high expression of OTUD7B and TRAF3 predicts good prognosis and that expression of these two proteins is positively correlated. Nonetheless, expression of NIK did not affect the prognosis of lung cancer patients (Fig. 8e).

In addition, based on data analysis at the starBase website (http://starbase.sysu.edu.cn), expression of NIK correlated significantly and positively with IL2 or MMP9 in lung cancer patients (Figure S2a, b), and analysis at the KM plotter website (http://kmplot.com) indicated that expression of NIK didn’t affect the prognosis of lung cancer patients (Figure S2c). Additionally, we found that patients with high expression of OTUD7B or TRAF3 had good prognosis (Figure S2d, e) and that expression of these two genes was positively correlated (Figure S2f). Further research through the cBioPortal website (http://www.cbioportal.org/) revealed a certain percentage of OTUD7B mutations in the population that may have adverse effects on prognosis (Figure S2g, h). The database analysis was consistent with our experimental findings.

## Discussion

LCL161 is an orally bioavailable Smac mimetic that induces apoptosis via IAPs degradation and caspase activation. Our previous study showed that LCL161 increases paclitaxel-induced apoptosis by degrading cIAP1 and cIAP2 in NSCLC [10]. In addition, LCL161 has been tested in phase I and phase II clinical trials and shown antitumor activity against leukaemia and ovarian, breast, colon, liver and lung cancers [25–27]. However, the function of Smac mimetics in cell invasion and migration remains controversial. Some studies support Smac mimetics inducing cancer cell elongation, invasion and migration [28, 29], and others report the opposite [30, 31]. In the present study, we found that LCL161 can induce lung cancer cell elongation, invasion and migration at a non-toxic concentration. In our previous study, we revealed that Smac mimetics degrade IAPs to activate caspase and promote apoptosis at a higher concentration. These effects might mask cell invasion and migration function. Thus, the contradictory results might be explained by the main functions occurring under different concentrations of the drug.

**Fig. 9:**
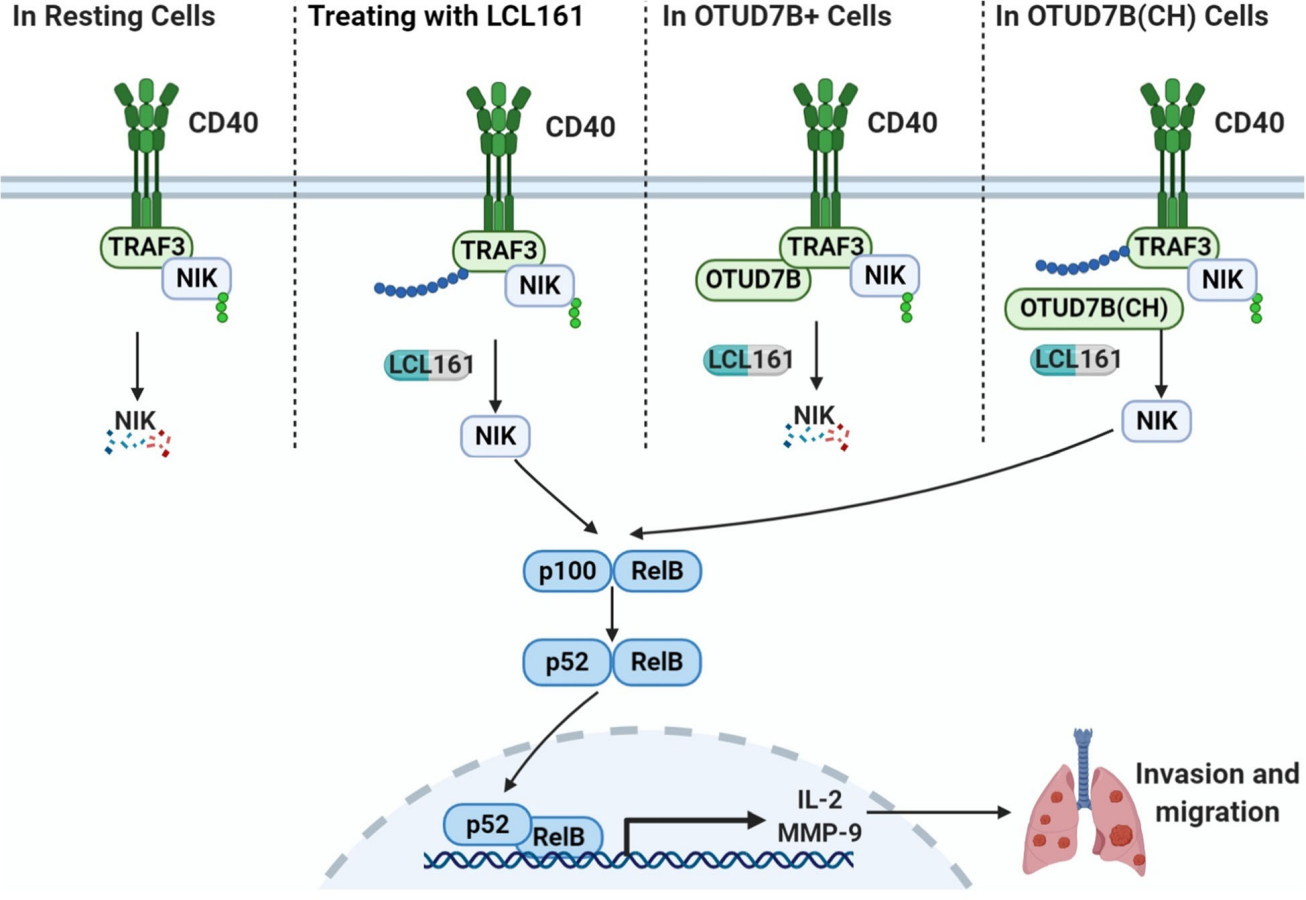
A model for OTUD7B deubiquitination of TRAF3 and inhibition of NIK to suppress LCL161-induced lung cancer cell invasion and migration. LCL161 activates the non-canonical NF-κB pathway via NIK accumulation and processing of p100 to p52, increasing the mRNA levels of NF-κB target genes to promote lung cancer cell invasion and migration. OTUD7B deubiquitinates TRAF3 and inhibits NIK and NF-κB pathways to suppress LCL161-induced cell invasion and migration. C194S/H358R(CH) in the OTU domain of OTUD7B and OTUD7B(CH) cannot deubiquitinate TRAF3 and regulate pathways such as wildtype OTUD7B can

We first determined the highest non-toxic concentration of LCL161 in lung cancer cells, at which there is no apparent effect on the apoptosis rate or viability but with degradation of cIAP1 and cIAP2 still being triggered. Then, we showed that LCL161 activated the non-canonical rather than canonical NF-κB pathway by causing NIK accumulation and p100 processing to p52. The mRNA levels of NF-κB target genes, such as MCP-1, IL-2, MMP-9, and CXCR-4, were significantly increased after treatment with LCL161. Obviously, overexpression of these genes is the direct reason for cell invasion and migration (Fig. 9). Furthermore, we showed that knockdown of NIK was able to suppress LCL161-induced lung cancer cell elongation, invasion and migration by inhibiting the non-canonical NF-κB pathway. According to database analysis, we found that expression of NIK correlated significantly and positively with IL-2 or MMP9 expression in lung cancer patients. In addition, expression of NIK did not affect the prognosis of lung cancer patients. Our study demonstrates that a nontoxic concentration of LCL161 can induce accumulation of NIK to promote invasion and metastasis but that an increase in NIK expression has no significant effect on the prognosis of lung cancer. Therefore, when toxic concentrations of LCL161 induce apoptosis, NIK accumulation does not necessarily have negative effects on patient prognosis. These findings in clinical analysis are in line with our experimental results.

Even if LCL161 promotes invasion only at low concentrations, an appropriate means of preventing this side effect should be found if it continues to be used it in clinical trials. As discussed above, the key component of LCL161-induced lung cancer cell invasion and migration is activation of the non-canonical NF-κB pathway. The deubiquitinases (DUBs) A20 and CYLD are important regulators of the canonical NF-κB pathway, but neither of them regulates the non-canonical NF-κB pathway [32]. Another DUB, OTUD7B, has a sequence homologous to A20 called the ovarian tumour (OTU) domain and is involved in the regulation of the non-canonical NF-κB pathway. A previous study showed that OTUD7B can inhibit the NF-κB pathway in liver cancer [33]. In our study, we found that overexpressed OTUD7B inhibits NIK and the non-canonical NF-κB pathway in lung cancer cells by binding to and deubiquitinating TRAF3. In this process, TRAF3 directly binds to NIK and leads to its degradation, eventually inhibiting the non-canonical NF-κB pathway (Fig. 9). In brief, OTUD7B deubiquitinates TRAF3 and inhibits NIK to suppress LCL161-induced lung cancer cell invasion and migration. This finding is meaningful. As LCL161 can enhance accumulation of NIK, which directly binds to TRAF3, none of these molecules are suitable for patient selection. Instead, OTUD7B is a good indicator for appropriately selecting patients for LCL161 treatment. A recent study showed that OTUD7B regulated invasion and migration via AKT pathway in lung cancer cells [34]. We found that there was no significant change in p-Akt and Akt after LCL161 treatment in both EV and OTUD7B overexpressed H1299 cells (Figure S1d). Although p-Akt was slightly increased after OTUD7B overexpression, it was not relative to cell migration and invasion caused by LCL161. Which is the dominant pathway for OTUD7B in cancer cell migration and invasion? Future experiments are needed to resolve this problem.

Lung cancer patients carry many gene mutations, including mutations in OTUD7B. Therefore, we need to determine which region of OTUD7B is necessary for inhibition of the non-canonical NF-κB pathway. A previous study reported that the OTU domain may be recognized as a DUB domain [35]. Therefore, we assume that OTUD7B-mediated TRAF3 deubiquitination requires DUB activity. We created a catalytically inactive mutation, C194S/H358R(CH), in the OTU domain of OTUD7B and found that OTUD7B(CH) cannot deubiquitinate TRAF3. Consistently, OTUD7B (WT) but not OTUD7B (CH) inhibited activation of the non-canonical NF-κB pathway and LCL161-induced lung cancer cell invasion and migration (Fig. 9). Accordingly, if LCL161 is used in the population with high OTUD7B expression, it is necessary to exclude the population with OTUD7B mutations in the OTU domain because this mutant may lead to a lack of NIK-mediated invasion and metastasis.

LCL161 is a promising drug that is being tested in early clinical trials and in combination with immune checkpoint inhibitors to treat cancer patients [36–38]. Although we are unaware of any reports about LCL161-induced invasion and migration in clinical trials, this is still a priority. The side effects of invasion and migration may be lethal in cancer patients, and it is difficult to distinguish these events from metastasis caused by disease progression.

## Conclusion

Our findings are the first to demonstrate that the non-canonical NF-κB pathway is the key mediator in LCL161-induced lung cancer cell invasion and migration. Furthermore, OTUD7B can inhibit LCL161-induced lung cancer cell invasion and migration by deubiquitinating TRAF3, inhibiting NIK and preventing non-canonical NF-κB activation. In addition, the OTU domain of OTUD7B is necessary for this regulation. Our study findings will contribute to the selection of lung cancer patients suitable for LCL161 treatment.

## Supplementary Information

**Additional file 1: Table S1** Primer sequences used in this study. **Table S2** Antibodies used in this study. **Table S3** Characteristics of NSCLC patients.

**Additional file 2: Figure S1.** Lung cancer cells were treated by LCL161 at a non-toxic concentration. (a) A549 and H1299 cells were treated for 24 h with the indicated concentration of LCL161 or DMSO. The apoptosis rate was detected by Annexin V-FITC/PI staining. (b) A549 and H1299 cells were treated with the indicated concentration of LCL161 or DMSO for 24 h. Expression of cIAP1, cIAP2, XIAP and activated caspase-3 was assessed by Western blotting. β-Actin served as the loading control. (c) A549 and H1299 cells were treated with the indicated concentration of LCL161 from 6 to 72 h or DMSO. Cell viability was detected by the MTT assay. (d) H1299 cells were treated with the indicated concentration of LCL161 or DMSO. Expression of p-Akt, Akt and OTUD7B was assessed by Western blotting. β-Actin served as the loading control.

**Additional file 3: Figure S2.** Analysis of expression of NIK, OTUD7B and TRAF3 in the clinical database. (a, b) The relationship between NIK expression and IL2 or MMP9 expression was analysed with lung adenocarcinoma patients data on the starBase website (http://starbase.sysu.edu.cn). (c, d, e) Kaplan–Meier analysis showed the relationship between lung cancer patient survival and NIK, OTUD7B, TRAF3 expression. The patient number at risk at different times of analyses is indicated at the bottom of the plots. The plots were generated using the KmPlot tool (http://www.kmplot.com/lung). Affymetrix ID 205192_at (NIK), 221571_at (TRAF3)_and 227436_at (OTUD7B) were used for analysis. (g, h) TCGA DNA sequencing results show that the OTUD7B gene is amplified and mutated at high frequencies in lung cancer patients (http://www.cbioportal.org/). The overall survival rate and disease-free survival rate of patients with or without the mutant OTUD7B gene are compared in the plot.

## Data Availability

All data generated or analysed during this study are included either in this article or in the supplementary information files.

## Abbreviations

NSCLC: Non-small cell lung cancer
LUSC: Lung squamous cell carcinoma
LUAD: Lung Adenocarcinoma
OTUD7B: OTU domain-containing 7B
TRAF3: TNF receptor-associated factor 3
NIK: NF-κB-inducing kinase
IAP: Inhibitor of apoptosis protein
XIAP: X-linked inhibitor of apoptosis protein
MMP: Matrix Metallopeptidase
MCP: Monocyte chemotactic protein
IL: Interleukin
CXCR: Chemokine receptor type
CYLD: Cylindromatosis
IHC: Immunohistochemistry
CHX: Cycloheximide
