# Supplementary material for: OTUD7B suppresses Smac mimetic-induced lung cancer cell invasion and migration via deubiquitinating TRAF3
Source: J Exp Clin Cancer Res. 2020 Nov 16;39:244. doi: 10.1186/s13046-020-01751-3 (PMC7667862; doi:10.1186/s13046-020-01751-3)
Supplement: Supplementary file 1 — Additional file 1: Table S1 Primer sequences used in this study. Table S2 Antibodies used in this study. Table S3 Characteristics of NSCLC patients. [file 13046_2020_1751_MOESM1_ESM.docx]

**Table S1 Primer sequences used in this study**

| Name | Primer (5'-3') |
| --- | --- |
| Human shNIK _F | GATCCCCTACCTCCACTCACGAAGGATTCAAGAGATCCTTCGTGAGTGGAGGTATTTTTGGAAA |
| Human shNIK_R | AGCTTTTCCAAAAATACCTCCACTCACGAAGGATCTCTTGAATCCTTCGTGAGTGGAGGTAGGG |
| Human IL2_qPCR_F | TCAACTCCTGCCACAATGTAC |
| Human IL2_qPCR_R | TGTAAATCCAGCAGTAAATGC |
| Human CXCR4_qPCR_F | GTGCCTTAGCCCACTACTTCAGA |
| Human CXCR4_qPCR_R | GGACATTCACTTCCAATTCAGCAA |
| Human MCP1_qPCR_F | GCTCATAGCAGCCACCTCATTC |
| Human MCP1_qPCR_R | CCGCCAAAATAACCGATGTGATAC |
| Human MMP2_qPCR_F | CAAGGACCGGTTTATTTGGC |
| Human MMP2_qPCR_R | ATTCCCTGCGAAGAACACAGC |
| Human MMP9_qPCR_F | TTGACAGCGACAAGAAGTGG |
| Human MMP9_qPCR_R | GCCATTCACGTCGTCCTTAT |
| Human TRAF3_qPCR_F | GCGCGCAAGCTTACCATGGAGTCGAGTAAAAAGATGG |
| Human TRAF3_qPCR_R | TGCCTGCTCGAGTCAGGGATCGGGCAGATCC |
| Human NIK_qPCR_F | GAGAATTCATGGCAGTGATGGAAATGG |
| Human NIK_qPCR_R | GCGGATCCTTAGGGCCTGTTCTCCAG |

**Table S2 Antibodies used in this study**

| Name | Catalogue number | Dilution | Supplier |
| --- | --- | --- | --- |
| OTUD7B | [16605-1-AP](https://www.ptglab.com/products/OTUD7B-Antibody-16605-1-AP.htm) | 1:500 | Proteintech |
| TRAF3 | 66310-1-Ig | 1:500 | Proteintech |
| NIK | Sc-8417 | 1:200 | Santa Cruz |
| p100 | 4810 | 1:500 | CST |
| p52 | 4882 | 1:500 | CST |
| RelB | 4954 | 1:500 | CST |
| c-IAP1 | 7065 | 1:1000 | CST |
| pIκBα | 2859 | 1:1000 | CST |
| IκBα | 9242 | 1:500 | CST |
| XIAP | ab21278 | 1:2000 | Abcam |
| HA | Sc-7392 | 1:1000 | Santa Cruz |
| Flag | 1804 | 1:1000 | Sigma |
| Myc | Sc-789 | 1:1000 | Santa Cruz |
| IL-2 | 26156-1-AP | 1:500 | Proteintech |
| MMP9 | ET704-69 | 1:200 | HuaBio |
| Ki67 | ST50-01 | 1:200 | HuaBio |
| p-Akt | sc-101629 | 1:500 | Santa Cruz |
| Akt | sc-5298 | 1:500 | Santa Cruz |
| Lamin B | sc-374015 | 1:500 | Santa Cruz |
| Tubulin | 2144 | 1:500 | CST |

**Table S3 Characteristics of NSCLC patients**

| Variable | No. of patients (%) |
| --- | --- |
| NSCLC patients | 146(100) |
| LUSC patients | 70(47.9) |
| LUAD patients | 76(52.1) |
| Age(years) |  |
| <70 | 98(67.1) |
| ≥70 | 48(32.9) |
| Sex |  |
| Male | 91(62.3) |
| Female | 55(37.7) |
| Smoking history |  |
| Former or current | 84(57.5) |
| Never | 62(42.5) |
| Tumour differentiation |  |
| Well | 62(42.5) |
| Moderately | 48(32.9) |
| Poorly | 36(24.6) |
| TNM stage |  |
| I | 51(34.9) |
| II | 55(37.7) |
| III | 40(27.4) |
| Lymphatic invasion |  |
| Present | 77(52.7) |
| Absent | 69(47.3) |
| Prognosis |  |
| Survival | 44(30.1) |
| Death | 102(49.9) |
