# Supplementary figures and images for: OTUD7B suppresses Smac mimetic-induced lung cancer cell invasion and migration via deubiquitinating TRAF3
Source: J Exp Clin Cancer Res. 2020 Nov 16;39:244. doi: 10.1186/s13046-020-01751-3 (PMC7667862; doi:10.1186/s13046-020-01751-3)

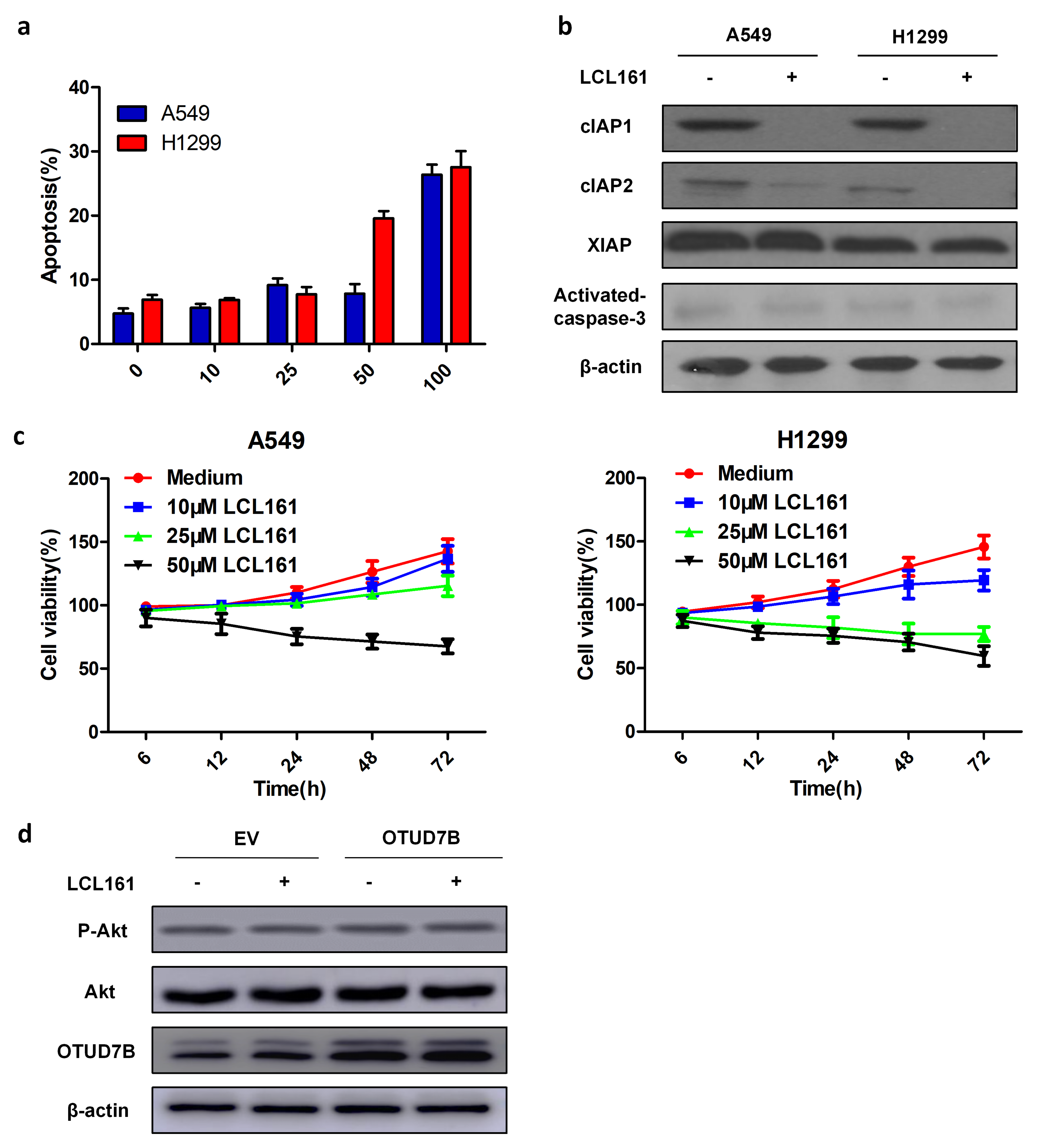

Supplement: Supplementary file 2 — Additional file 2: Figure S1. Lung cancer cells were treated by LCL161 at a non-toxic concentration. (a) A549 and H1299 cells were treated for 24 h with the indicated concentration of LCL161 or DMSO. The apoptosis rate was detected by Annexin V-FITC/PI staining. (b) A549 and H1299 cells were treated with the indicated concentration of LCL161 or DMSO for 24 h. Expression of cIAP1, cIAP2, XIAP and activated caspase-3 was assessed by Western blotting. β-Actin served as the loading control. (c) A549 and H1299 cells were treated with the indicated concentration of LCL161 from 6 to 72 h or DMSO. Cell viability was detected by the MTT assay. (d) H1299 cells were treated with the indicated concentration of LCL161 or DMSO. Expression of p-Akt, Akt and OTUD7B was assessed by Western blotting. β-Actin served as the loading control. [file 13046_2020_1751_MOESM2_ESM.tif]

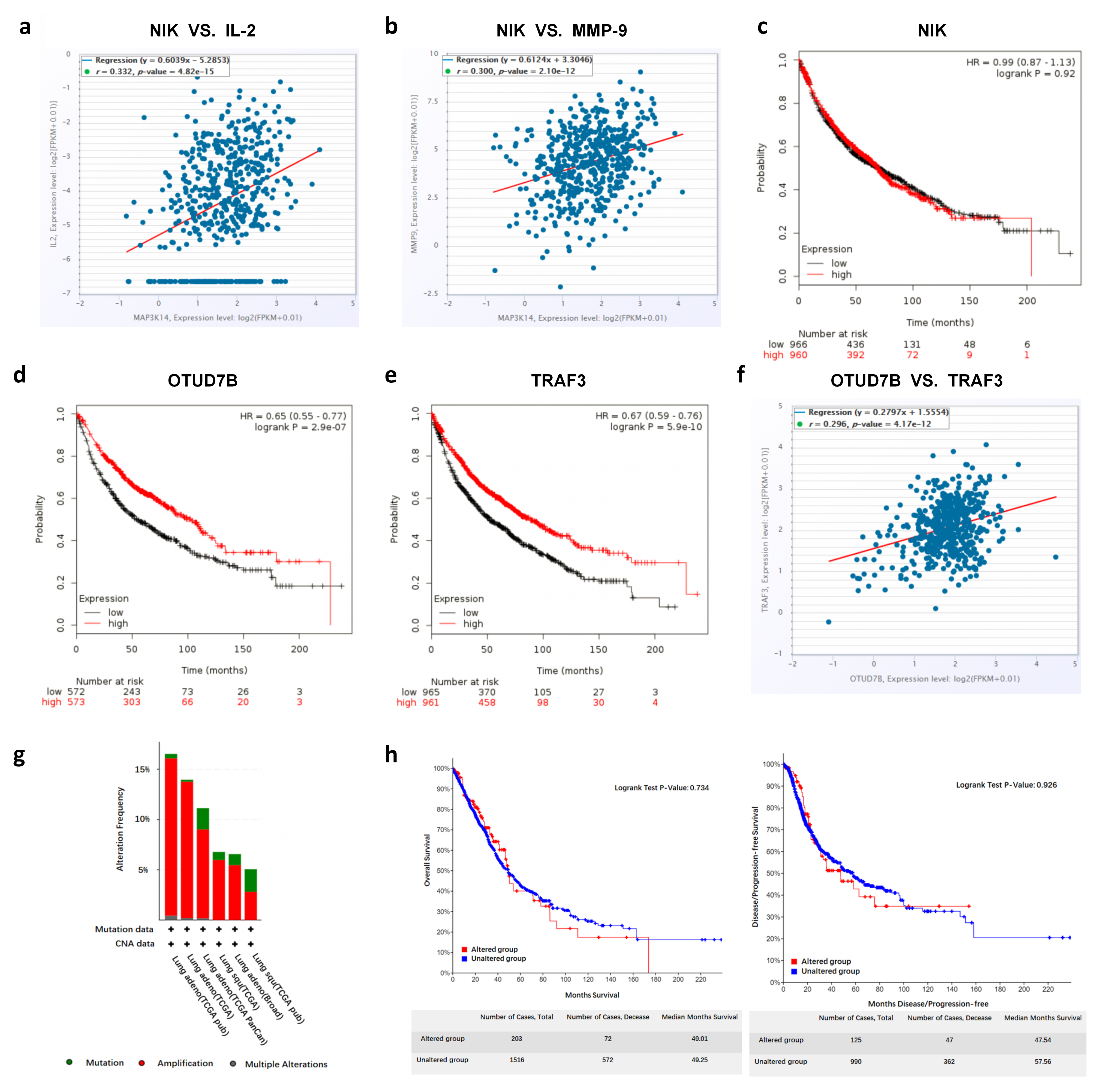

Supplement: Supplementary file 3 — Additional file 3: Figure S2. Analysis of expression of NIK, OTUD7B and TRAF3 in the clinical database. (a, b) The relationship between NIK expression and IL2 or MMP9 expression was analysed with lung adenocarcinoma patients data on the starBase website (http://starbase.sysu.edu.cn). (c, d, e) Kaplan–Meier analysis showed the relationship between lung cancer patient survival and NIK, OTUD7B, TRAF3 expression. The patient number at risk at different times of analyses is indicated at the bottom of the plots. The plots were generated using the KmPlot tool (http://www.kmplot.com/lung). Affymetrix ID 205192_at (NIK), 221571_at (TRAF3)_and 227436_at (OTUD7B) were used for analysis. (g, h) TCGA DNA sequencing results show that the OTUD7B gene is amplified and mutated at high frequencies in lung cancer patients (http://www.cbioportal.org/). The overall survival rate and disease-free survival rate of patients with or without the mutant OTUD7B gene are compared in the plot. [file 13046_2020_1751_MOESM3_ESM.tif]
